# Neutrophilia and NETopathy as Key Pathologic Drivers of Progressive Lung Impairment in Patients With COVID-19

**DOI:** 10.3389/fphar.2020.00870

**Published:** 2020-06-05

**Authors:** Teluguakula Narasaraju, Benjamin M. Tang, Martin Herrmann, Sylviane Muller, Vincent T. K. Chow, Marko Radic

**Affiliations:** ^1^College of Veterinary Medicine, Oklahoma State University, Stillwater, OK, United States; ^2^Department of Intensive Care Medicine, Nepean Hospital, Sydney, NSW, Australia; ^3^Department of Internal Medicine 3, Universitätsklinikum Erlangen, Friedrich-Alexander Universität Erlangen-Nürnberg, Erlangen, Germany; ^4^CNRS-University of Strasbourg, Biotechnology and Cell Signaling, Illkirch, France; ^5^Laboratory of Excellence Medalis, Institut de science et d'ingénierie supramoléculaire, and University of Strasbourg Institute for Advanced Study (USIAS), Strasbourg, France; ^6^Department of Microbiology and Immunology, Infectious Diseases Program, School of Medicine, National University of Singapore, National University Health System, Singapore, Singapore; ^7^Department of Microbiology, Immunology and Biochemistry, College of Medicine, University of Tennessee Health Science Center, Memphis, TN, United States

**Keywords:** COVID-19, SARS-CoV-2, acute respiratory distress syndrome, neutrophils, neutrophilia, neutrophil extracellular traps, pathogenesis, therapeutics

## Abstract

There is an urgent need for new therapeutic strategies to contain the spread of the novel coronavirus disease 2019 (COVID-19) and to curtail its most severe complications. Severely ill patients experience pathologic manifestations of acute respiratory distress syndrome (ARDS), and clinical reports demonstrate striking neutrophilia, elevated levels of multiple cytokines, and an exaggerated inflammatory response in fatal COVID-19. Mechanical respirator devices are the most widely applied therapy for ARDS in COVID-19, yet mechanical ventilation achieves strikingly poor survival. Many patients, who recover, experience impaired cognition or physical disability. In this review, we argue the need to develop therapies aimed at inhibiting neutrophil recruitment, activation, degranulation, and neutrophil extracellular trap (NET) release. Moreover, we suggest that currently available pharmacologic approaches should be tested as treatments for ARDS in COVID-19. In our view, targeting host-mediated immunopathology holds promise to alleviate progressive pathologic complications of ARDS and reduce morbidities and mortalities in severely ill patients with COVID-19.

## Introduction

The recent coronavirus disease 2019 (COVID-19) pandemic that started in Hubei Province of China has spread rapidly around the world (Wu J. et al., 2020; Zhu et al., 2020). Although COVID-19 related deaths were mainly reported in China until mid-February 2020, by late March, the virus spread globally with sharp increases in fatal infections in most countries especially Iran, Italy, South Korea, Spain, and USA (Hoseinpour Dehkordi et al., 2020). As of May 22, 2020, WHO has documented about five million COVID-19 cases and over 330,000 deaths worldwide, with an estimated case fatality rate of over 6.5% (W.H.O. Rolling Updates on COVID-19). COVID-19 is caused by SARS-coronavirus-2 (SARS-CoV-2), which belongs to the coronavirus family and is related to severe acute respiratory syndrome (SARS) and Middle East respiratory syndrome (MERS) viruses. Structurally, SARS-CoV-2 is an enveloped virus with a positive-sense single-stranded RNA genome (Lu et al., 2020). The spike proteins on the virion surface are responsible for engaging the angiotensin converting enzyme 2 (ACE2) receptor for entry into susceptible host cells; the initial infection predominantly targets epithelial cells of lungs and pharynx (Zhang H. et al., 2020). Aerosolized droplets are thought to be the primary viral transmission mode, (van Doremalen et al., 2020) and asymptomatic carriers are a significant source of community spread (Zhang J. et al., 2020). There are currently no vaccines nor antiviral drugs available for routine use to prevent and treat COVID-19. Rapidly, clinical trials have been initiated, but effective vaccines and drugs will likely take many months to reach the global market.

Current NIH treatment guidelines recommend the use of remdesivir in the treatment of patients with severe COVID-19 and advise against the use of HIV protease inhibitors lopinavir and ritonavir (COVID-19 Treatment Guidelines Panel). Secondary infections are treated with combinations of antibiotics, and glucocorticoids may be recommended, based on the clinical condition of the patients, if assisted ventilation is required (Wang et al., 2020; Wu C. et al., 2020). At the current magnitude of contagious infections and associated deaths caused by COVID-19, there is urgency to employ additional strategies and treatment options to reduce fatalities. To design novel treatments, a better understanding of COVID-19 pathogenesis is essential, especially as it pertains to crucial host-pathogen interactions. Given that serum levels of the proinflammatory cytokine IL-6 are significantly elevated in patients with severe COVID-19, clinical trials have been initiated to evaluate antibodies that block IL-6 or IL-6 receptor such as tocilizumab (Actemra, Roche; currently in 35 Clinical Trials, e.g., NCT04317092) and sarilumab (Kevzara, Regeneron; currently in 12 Clinical Trials, e.g., NCT04315298). Although initial results appear encouraging (Xu X. et al., 2020), there remains a need for other strategies, particularly for cell-targeted approaches.

### Neutrophilia Is Associated With Fatal COVID-19 Infections

Patients with COVID-19 develop clinical manifestations of high fever, cough, myalgia, dyspnea, and pneumonia. Significant numbers of hospitalized patients with respiratory symptoms eventually develop severe to critical illness with progressive clinical manifestations of acute respiratory distress syndrome (ARDS) before succumbing to infection (Chen et al., 2020; Huang et al., 2020; Pan et al., 2020; Zhou et al., 2020). In addition to direct virus-inflicted pathologies, exaggerated immune responses resulting in a “cytokine storm” contribute to disease severity in subgroups of patients with advanced disease (Chen et al., 2020; Huang et al., 2020; Mehta et al., 2020; World Health Organization; Zhou et al., 2020).

The most significant clinical finding in patients who require management in the intensive care (ICU) is fulminant neutrophilia (Huang et al., 2020; Zhou et al., 2020). Patients with COVID-19, who were admitted to the ICU, had greatly increased blood neutrophil counts as compared to other SARS-CoV2-positive patients with less severe symptoms (Huang et al., 2020). The neutrophil counts increase in parallel with severity of disease, indicating that an elevated neutrophil-lymphocyte ratio could be an early prognostic marker in COVID-19 infections (Gong et al., 2020; Zhang B. et al., 2020). Yang et al. (2020). found that critically ill patients develop high neutrophilia before succumbing to infection, thus confirming the association between excessive neutrophil loads and acute lung pathology in fatal COVID-19.

Histopathology of lung biopsies and autopsy specimens demonstrated that patients infected with SARS-CoV-2 develop manifestations of ARDS with abundant pathologic lesions, including prominent bronchopneumonia and peribronchial cuffing with neutrophils and histocytes, denuded alveolar epithelium, widespread hemorrhagic effusions, fibrin deposition, and protein exudates that fill alveolar air sacs. Interestingly, abnormal tissue remodeling with proliferating epithelium are also observed in COVID-19 infected patients (Barnes et al., 2020; Barton et al., 2020; Zhang H. et al., 2020) ARDS has previously been identified in acute respiratory infections with SARS and MERS and, notably, in patients with severe influenza (Lew et al., 2003; Kim et al., 2016; Huang et al., 2018). Indeed, enhanced neutrophilia has been directly correlated with disease severity in influenza-infected patients (Ishigaki et al., 2011). Increased serum levels of neutrophil enzymes serve as markers for poor prognosis in patients with severe influenza pneumonia (Zhu et al., 2018). Moreover, pathway analysis of the blood transcriptome identified a “neutrophil-dominated” immune response that clearly and unambiguously connects severe influenza that requires ICU admission to the activation of circulating neutrophils in this disease (Dunning et al., 2018; Tang et al., 2019). An initial analysis of COVID-19-derived bronchoalveolar lavage (BAL) and peripheral blood mononuclear cell (PBMC) RNA transcriptomes has recently been reported (Xiong et al., 2020), but there remains urgent need to carry out additional transcriptomics analyses in patients with COVID-19.

A ground-breaking study on neutrophil activation products in sera from patients with severe COVID-19 places neutrophils at the center of ARDS pathogenesis (Zuo et al., 2020). Neutrophils were traditionally viewed as short-lived and terminally differentiated innate immune cells that function as primary responders against infection or injury. More recent molecular analyses have uncovered an astounding degree of neutrophil heterogeneity and plasticity that are most vividly displayed during infections, in autoimmunity, or in oncology (Silvestre-Roig et al., 2016). In response to myriad external stimuli, neutrophils release potent enzymes such as neutrophil elastase (NE) and myeloperoxidase (MPO) from cytoplasmic granules (Borregaard et al., 2007). Although neutrophils contribute to host immunity, excessive recruitment of neutrophils and their release of granule components along with nuclear chromatin, or neutrophil extracellular traps (NETs), (Brinkmann et al., 2004) aggravate tissue injury and may lead to death in several disease conditions (Xu et al., 2009; Abrams et al., 2013; Sivanandham et al., 2018). NETs lead to a dispersal of toxic molecules: histones and granule proteins such as MPO, NE, and proteinase 3 (Narasaraju et al., 2011; Ashar et al., 2018; Zhu et al., 2018), and, importantly, NETs strongly stimulate the production of pro-inflammatory cytokines (Muller and Radic, 2016). Released NETs can disrupt alveolar epithelium and endothelium, and also degrade the thin alveolar basement membrane, culminating in epithelial necrosis, denudation of epithelial lining, vascular damage, pulmonary edema, and hemorrhage in lethal influenza-infected mice (Narasaraju et al., 2011; Ashar et al., 2018; Zhu et al., 2018). In addition, at high neutrophil densities, NETs tend to form large aggregates that can be a significant source of enzymatic activities (Schauer et al., 2014), which may accelerate the formation of thrombi in blood vessels during an infection (Boeltz et al., 2017). In COVID-19 pathogenesis, lung infection may accelerate local thromboembolic events, for which neutrophil activity may contribute an essential component (Ciceri et al., 2020; Klok et al., 2020).

**Figure 1:**
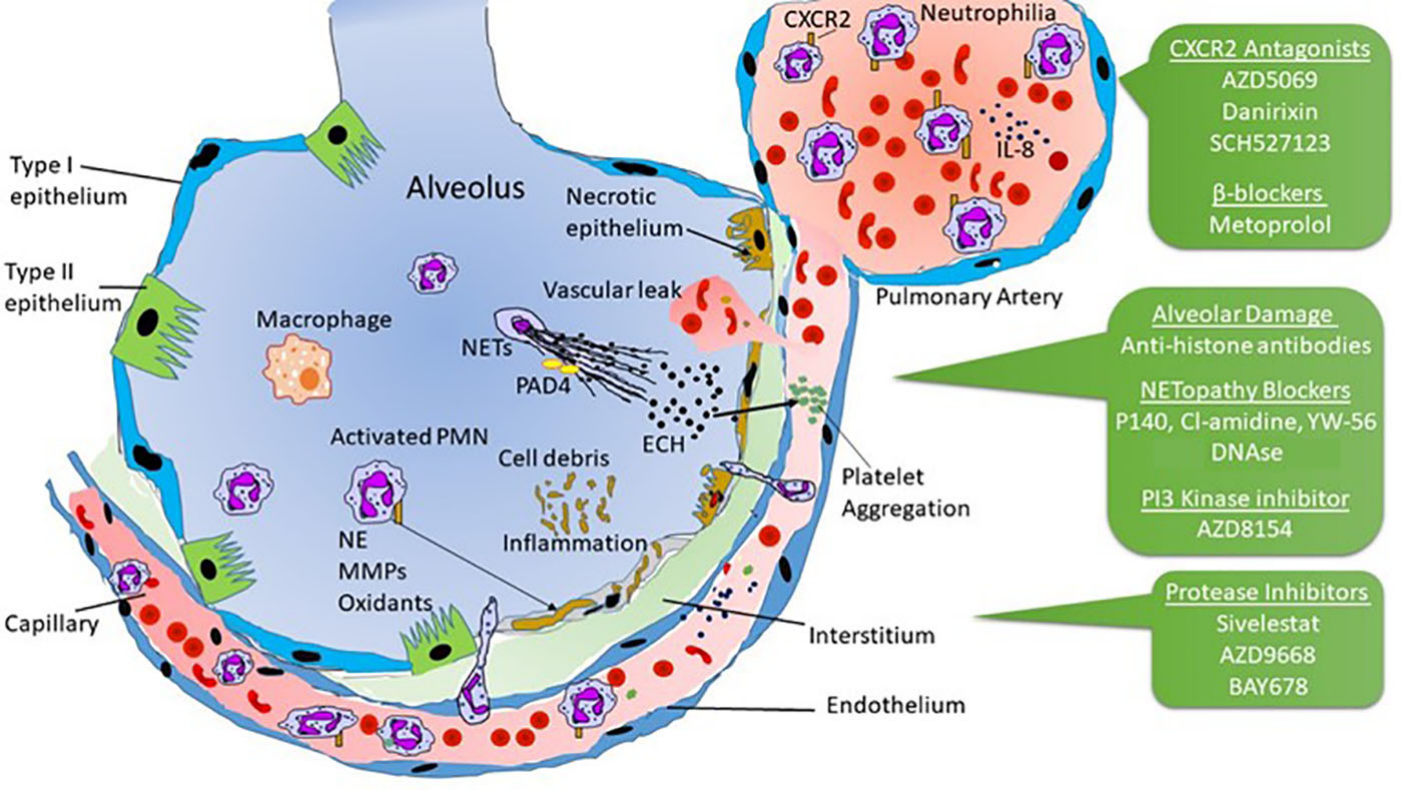
Possible mechanisms of neutrophilia and NETopathy in alveolar-capillary damage affecting lungs in severe COVID-19. Pharmacological agents that may find applications in progressive lung pathology are indicated in the green boxes and discussed in the text.

Accordingly, in severe COVID-19, greater neutrophilia may drive elevated pulmonary influx of neutrophils and stimulate excessive NET release, which can exacerbate alveolar-capillary damage and lead to pathologic manifestation of ARDS (**Figure 1**). The released NET chromatin, containing large amounts of extracellular histones, can disrupt epithelial lining and induce platelet aggregation leading to pulmonary vascular thrombosis. Over time, the accumulated cellular debris may precipitate inflammation and magnify the cytokine storm (Pedersen and Ho, 2020). This scenario is consistent with the detection of elevated NET breakdown products in the sera of severely ill patients with COVID-19 (Zuo et al., 2020). Clearly, detailed analyses of BAL for indicators of neutrophil activation and NET release are urgently needed to determine if neutrophilic influx, and NETosis are important drivers of progressive pulmonary pathology in the most severe cases of COVID-19.

A note of caution must be included with this synopsis. Current emergency treatments for patients with severe complications of COVID-19 usually require the use of external lung ventilator devices. However, the use of ventilators may present its own peril. Notably, NET markers are increased in alveolar spaces of patients with ventilator-associated pneumonia (VAP) (Mikacenic et al., 2018) and NETs can be detected in mouse models of mechanical ventilation (Yildiz et al., 2015; Li et al., 2017). Thus, the use of mechanical respirators for severe COVID-19 infections may need monitoring according to neutrophil counts at the beginning and throughout assisted respiration. Conversely, lessons learned during this pandemic health emergency will hopefully inform future applications of this technology in critically ill patients.

### Targeting Neutrophils and NET-Associated Lung Injury in Patients With Severe COVID-19

Administration of drugs that prevent neutrophil recruitment and activity may effectively attenuate the pathologic complications of alveolitis and vascular injury in patients with COVID-19 (**Figure 1**), and initial results of several treatment strategies have been reported (COVID-19 Treatment Guidelines Panel). One early randomized clinical trial was the infusion of vitamin C in patients with COVID-19, based on the hypothesis that vitamin C suppresses neutrophil influx, activation, and NET-associated alveolar-capillary damage (Peng, NCT04264533). Although vitamin C exhibits anti-oxidant function, its effect on reducing neutrophil activity and NETs-mediated pathologies *in vivo* may be limited. In contrast, small molecules and peptides may reduce NET release in important ways. This is the case of the therapeutic peptide P140, which has been tested in clinical trials (Zimmer et al., 2013) and prevents NET release *in vitro* (Bendorius et al., 2018). This peptide regulates chaperone-mediated autophagy and macroautophagy (Macri et al., 2015), thereby reducing excessive inflammation that is a prominent feature in patients with autoimmune disorders. The effect of P140 on the neutrophil influx in the bronchoalveolar space requires specific attention.

### Inhibitors of Neutrophil Migration and Activation

Neutrophil influx into the lung parenchyma requires chemoattraction, binding to endothelial receptors, signaling, and transmigration. These steps are the focus of many *in vivo* and *in vitro* studies, and pharmacological inhibitors are available to address these separate stages of neutrophil migration and activation. An important chemokine in infection and inflammation is CXCL-8/IL-8. Binding of IL-8 to CXCR2 on neutrophils activates neutrophils and leads to NET release (Tatsiy and McDonald, 2018). Interestingly, a wide range of CXCR2 chemokine receptor agonists induce NETs *in vivo* (Teijeira et al., 2020). Therefore, targeted inhibition of neutrophil activation and NET release may be accomplished using antagonists of the neutrophil chemokine receptor CXCR2.

Small molecule CXCR2 antagonists have been extensively tested in clinical trials for asthma, chronic obstructive pulmonary disease (COPD) and influenza. AZD5069 (AstraZeneca) is a selective CXCR2 antagonist that has been tested for safety and efficacy in pre-clinical and clinical studies of COPD and asthma (O'Byrne et al., 2016; Pedersen et al., 2018). AZD5069 was able to block neutrophil trafficking while preserving neutrophil-mediated host immunity (Jurcevic et al., 2015; Uddin et al., 2017). The administration of AZD5069 reduced NETopathic inflammation of sputum neutrophils from patients with COPD (Pedersen et al., 2018; Uddin et al., 2019). Similarly, the CXCR2 inhibitor Danirixin (GlaxoSmithKline) has been tested in phase 2 clinical trials in patients with COPD and influenza, where it reduced neutrophilia (Madan et al., 2019; Roberts et al., 2019). A third CXCR2 antagonist, SCH527123 (Merck), inhibited lung neutrophil influx in asthma patients, and decreased neutrophilia in healthy humans exposed to toxic levels of ozone (Holz et al., 2010; Nair et al., 2012). Although these selective CXCR2 antagonists decreased neutrophilia in chronic respiratory diseases, their efficacy in COVID-19 and other acute lung infections needs to be urgently tested.

Chemokine signals induce neutrophil attachment to endothelia by activating integrin adhesion receptors and enhancing integrin binding to the actin cytoskeleton, which are mediated by PI3 kinase (Yago et al., 2018). The dual inhibitor of the delta and gamma subunits of PI3 kinase, AZD8154, which is currently in a phase I trial for asthma (Clinical Trials, NCT04187508), inhibits the initial step in neutrophil extravasation and thus may offer therapeutic benefit in lung pathology associated with severe COVID-19.

### Inhibitors of Neutrophil Proteases

A distinct class of drugs that may potentially ameliorate alveolitis in COVID-19 are neutrophil protease inhibitors. Histopathologic examination of lung tissues from deceased patients with COVID-19 reveals interstitial fibrosis, chronic inflammation, and formation of intra-alveolar fibrous plugs (Xu Z. et al., 2020). These findings indicate possible abnormal lung remodeling and degeneration due to augmented neutrophil-derived protease activity. NE is a protease capable of degrading multiple protein targets, including extracellular matrix proteins such as elastin, collagen and fibronectin, which are abundant proteins of the alveolar basement membrane. NE inhibitors, e.g., sivelestat sodium (ONO Pharmaceuticals), have been evaluated in clinical trials in patients with COPD (Hayakawa et al., 2010; Morjaria et al., 2010), and in acute lung injury patients (Yoshikawa et al., 2010). The use of sivelestat together with oseltamivir effectively reduced lung injury in a patient infected with the 2009 pandemic swine-influenza virus (Yokoyama et al., 2010). Other NE inhibitors, such as AZD9668 (AstraZeneca) and BAY-678 (Bayer), have undergone clinical trials for the treatment of COPD (Gunawardena et al., 2013; von Nussbaum et al., 2015), and thus may provide potential alternative therapies to prevent alveolar-capillary damage in patients with COVID-19. Interestingly, the enzymatic activity of NE is quenched by binding to NETs (Podolska et al., 2019).

### Inhibitors of Peptidylarginine Deiminase IV

More direct inhibitors of NET release target peptidylarginine deiminase IV (PAD4). This enzyme converts arginine to citrulline in cellular substrates, including core histones, which promotes chromatin unwinding and NET release (Neeli et al., 2008). PAD4 inhibitors, including Cl-amidine, YW-56, and GSK484, have shown efficacy in NET-mediated pathologies, including in animal models of thrombosis associated with myeloproliferative neoplasms (Wolach et al., 2018), lethal lung endotoxemia (Liang et al., 2018), and cellular damage due to hypoxia (Lange et al., 2014). Administration of these inhibitors, either systemically or directly into the lungs, may benefit from the simultaneous dispersal of NETs. Clearly, clinical trials to assess the use of neutrophil recruitment blockers, protease inhibitors or NETosis suppressors in the management of severely ill patients with COVID-19 should be considered as potential therapeutic strategies to reduce the progressive lung pathology and its possible fatal outcome.

### Recombinant DNase-1

A currently available therapeutic that degrades DNA and dissolves NETs is recombinant DNase (Pulmozyme, Genentech), which may allow better penetration of co-administered compounds into affected lungs (Cortjens et al., 2018). However, circulating DNase-1-generated NET degradation products still possess pro-inflammatory capabilities as well as protease activity (Schauer et al., 2014). This bears the risk of spreading inflammation and enlarging the areas of tissue damage. It stands to reason that the effect of recombinant DNase on thrombo-inflammation in COVID-19 will require further studies.

### β-Blockers

Selective inhibition of adrenergic signaling by the β1-blocker metoprolol in patients with myocardial infarction causes “stunning” of neutrophils and reduces the infarct size (García-Prieto et al., 2017). In addition to its effect on cardiomyocytes, metoprolol inhibits neutrophil migration. It acts early in the process of neutrophil recruitment by reducing the engagement of activated circulating platelets and thus ameliorates inflammation (García-Prieto et al., 2017). Interestingly, metoprolol also inhibits the NET-mediated pathology in the gall bladder (Munoz et al., 2019).

### Additional Immunopathologic Complications May Contribute to Severe COVID-19

Clinical reports document that about one in five SARS patients suffer complications of bronchopneumonia, with superinfection by bacterial and other pathogens (Ng et al., 2006). Hence, it is likely that a substantial fraction of patients with severe COVID-19 (including those with pneumonia arising from assisted ventilation) experience secondary bacterial pneumonia associated with excessive neutrophil infiltration and NETs within infected lung tissues.

Remarkably, an increase in fibrin degradation products (D-dimers) has been linked to fatal COVID-19 infections (Gong et al., 2020; Wang et al., 2020). Increased coagulation due to elevated D-dimers may instigate occlusion of small blood vessels in pulmonary vasculature that leads to ischemia, vascular injury, and hemorrhagic effusions in critically ill patients. Widespread pulmonary vascular thrombosis has also been documented in severe influenza, including during the 1918 Spanish flu and the 2009 swine-flu pandemics, especially in autopsy findings (LeCount, 1919; Walters et al., 2016). This type of pathology may also be attributed to secondary bacterial pneumonia (Morens et al., 2008; Rudd et al., 2016). Studies reveal that extracellular histones released from NETs lead to alveolar-capillary damage and potentially mediate platelet activation, aggregation, and development of vascular thrombosis (Xu et al., 2009; Abrams et al., 2013; Ashar et al., 2018). Platelet activation may stimulate further NET release, thus engendering a vicious cycle (Maugeri et al., 2014). Interestingly, increased fibrinogen degradation products (FDP), indicators of heightened thrombotic status, are also observed in animal models of influenza, where the use of anti-histone antibodies could suppress alveolitis and vascular thrombosis (Ashar et al., 2018). Furthermore, in conditions of murine neutrophilia, intravascular formation and aggregation of NETs may form thrombi that are often fatal, especially in the context of low activities of the serum-borne DNase-1 and DNase-1L3 (Jiménez-Alcázar et al., 2017).

Highly notable pathologic features common to COVID-19, SARS, and secondary bacterial pneumonia are the prominent proteinaceous exudates in alveolar spaces, i.e., pulmonary edema that contributes to ARDS. These are attributed to increased vascular permeability associated with severe inflammation, which may be a direct effect of neutrophil extravasation. Neutrophils exit the vasculature and reach the alveolar spaces by disrupting tight junctions between endothelial cells through the action of proteinase 3 (Kuckleburg et al., 2012). Under extreme conditions, stress on the endothelial junctions may result in fluid leakage into the lungs.

### Future Therapeutic Choices

A better understanding of COVID-19 immunopathology is critical to develop novel therapeutic interventions. A recent histopathologic analysis of the lung biopsy of a COVID-19 patient identified diffuse alveolar damage, necrotic epithelium, epithelial denudation, with large numbers of epithelial cells in the alveolar air space (Zhang H. et al., 2020). Temporal changes in inflammatory cytokine responses in BAL may identify factors that contribute to pathogenic changes in ARDS and uniquely distinguish critically ill patients. Analysis of BAL for increased levels of NE, matrix metalloproteinases (MMPs), and the accumulation of deiminated histones could become essential diagnostic tools to rapidly assess different target strategies to attenuate pathogenic progression in ICU patients with severe pulmonary complications. It is important to point out that lung function may be compromised by various other etiologies or their complications, as it is unlikely that a single pathogenic mechanism operates in all affected patients. In particular, any intervention designed to inhibit neutrophil functions must be weighed carefully against the fact that adequate innate immunity is required in any infectious disease, especially COVID-19, where a secondary bacterial or fungal infection may arise during prolonged intubation. Nevertheless, greater attention on neutrophils may help lessen the burden of COVID-19 in the severely ill.

## Author Contributions

TN, MR, VC, MH, and SM drafted the manuscript and revised the final form. BT provided key insights for the manuscript. All authors contributed to the article and approved the submitted version.

## Conflict of Interest

The authors declare that the research was conducted in the absence of any commercial or financial relationships that could be construed as a potential conflict of interest.
